# Evaluating the Translation Value of Two *In Vivo* Models for Breast Cancer Brain Metastases

**DOI:** 10.3390/cancers18071095

**Published:** 2026-03-27

**Authors:** Sigrid Cold, Maria Zeiler Alfsen, Brandur Halgirsson, Mads Neergaard Jorgensen, Jacob Hald, Carsten Haagen Nielsen, Andreas Kjaer, Lotte Kellemann Kristensen, Trine Bjornbo Engel

**Affiliations:** 1 Minerva Imaging ApS, 3650 Ølstykke, Denmark; sco@minervaimaging.com (S.C.);; 2 Cluster for Molecular Imaging, Department of Biomedical Sciences, University of Copenhagen, 2200 Copenhagen, Denmark; 3 Department of Clinical Physiology and Nuclear Medicine, Copenhagen University Hospital, Rigshospitalet, 2100 Copenhagen, Denmark

**Keywords:** brain metastasis, orthotopic model, intracarotid inoculation, imaging, breast cancer

## Abstract

Background: Breast cancer brain metastases (BCBM) lack effective treatments, contributing to breast cancer-related morbidity and mortality. Integrating translational animal models and advanced non-invasive imaging can accelerate the development of urgently needed therapies. Method: In this study, we developed an intracarotid method mimicking BCBM and compared it to the stereotactic model in terms of animal welfare, tumour establishment, and blood–brain barrier (BBB) permeability. BCBM was established through intracarotid or stereotactic inoculation of BT474 and MDA-MB-231.Luc2 cells in NMRI nude mice. We utilised magnetic resonance imaging (MRI) and bioluminescence imaging (BLI) to monitor tumour growth and BBB permeability, supported by fluorescent immunohistochemistry for validation. Finally, light sheet microscopy (LSM) was employed to visualise tumour establishment in intact brains. Results: Both inoculation methods achieved a survival rate > 70%, with animals recovering within a week post-surgery. MRI and BLI effectively visualised tumour growth with stereotactic implantation, resulting in single tumours, while intracarotid inoculation led to micro-seeding of up to seven tumours in one brain. Tumour growth was rapid and homogenous in the stereotactic model, whereas the intracarotid model exhibited slower, heterogenous growth. Notably, BBB permeability was significantly higher in small tumours in the stereotactic model when compared to the intracarotid model (*p* = 0.003). Ex vivo analyses validated these findings with the identification of multiple metastasis in the intracarotid model and single tumours in the stereotactic model. Conclusions: We developed an animal model that closely mimics BCBM, highlighting extravasation and micro-seeding while maintaining animal welfare. Our established imaging protocols enable longitudinal evaluations of BBB permeability and treatment response, creating a translational platform for testing novel anti-cancer therapies.

## Simple Summary

Translational animal models of breast cancer brain metastasis (BCBM) support the development of urgently needed therapies. Today, the most commonly used murine model is generated by stereotactic implantation of breast cancer cells into the mouse brain. While the model is reproducible and reliable, it lacks several important aspects of BCBM, including extravasation of cancer cells from the blood into the brain and micro-seeding. In this study, we established a reproducible BCBM model by injecting breast cancer cells into the carotid artery of mice and evaluated the model in terms of operational success, animal welfare and tumour establishment against the stereotactic model. The intracarotid model was found to mimic BCBM to a greater extent than the stereotactic model, with the establishment of multiple metastasis in the brain. Furthermore, a difference in the blood–brain barrier permeability was observed between the two models, a critical factor for drug delivery to intracranial lesions.

## 1. Introduction

Breast cancer (BC) is the most diagnosed cancer in women worldwide, with over 2.3 million new cases in 2022. Despite advancements in screening, early diagnosis, and treatment, more than 670,000 BC patients die annually [1]. The progression of localised BC into metastatic disease is the primary cause of BC-related morbidity and death [2]. Metastatic BC affects up to 30% of patients, with bone, liver, lung and brain being common sites of metastasis [3]. BC brain metastasis (BCBM) is the most lethal type of BC metastasis, affecting more than 14% of patients with metastatic disease [4]. The risk of developing BCBM is especially high in patients with human epidermal growth factor receptor 2 (HER2)-expressing BC or triple-negative BC (TNBC) [5,6,7]. Patients with BCBM have a poor prognosis, with a median survival time of two to nine months despite receiving therapy, highlighting the urgent need for new treatments [8].

Drug candidates showing promising preclinical results often fail in a clinical setting [9]. Drugs targeting intracranial tumours or metastases have proven especially challenging, with very low success rates [10,11,12]. Preclinical testing of novel therapies is often conducted in animals bearing subcutaneous tumours, which simplifies monitoring and efficacy evaluation [13]. However, these models fail to resemble the tumour microenvironment, which has been shown to play a pivotal role in tumour establishment, growth, and treatment response [14]. Orthotopic models, where tumours are established in the organ of origin, offer greater translational value by allowing therapy evaluation in a more biologically relevant context [15]. The tumour microenvironment of BCBM includes the blood–brain barrier (BBB), which, along with the lack of translational animal models, poses significant challenges in developing BCBM therapies [16,17,18]. The BBB restricts the delivery of larger biologics and small molecule compounds [19,20]. During tumour growth, cancer cell interaction with the BBB leads to heterogenous BBB disruption, allowing limited delivery of compounds usually restricted from the brain [21]. Developing animal models that mimic the clinical BBB disruption observed in patients with BCBM is crucial for effective drug development and, ultimately, patient survival.

Most BC therapies have minimal effect on BCBM, partly due to the BBB [22]. Preclinical evaluation of therapies targeting BCBM is often conducted in an orthotopic model of BCBM, established by stereotactic implantation of BC cells through the skull directly into the brain tissue. This model is reproducible and incorporates the microenvironment of intracranial tumours but results in a single tumour at a predefined location, resembling primary brain tumours more than metastatic disease [23]. In contrast, metastatic diseases involve extravasation of cancer cells from the bloodstream to distant organs, resulting in micro-seeding of multiple individual metastases [24].

More elaborate orthotopic models for BCBM involve injecting cells into the bloodstream. Tail vein and intracardial injections are easy but often lead to metastasis in highly perfused organs such as lungs and liver before brain metastases [25,26]. Carotid artery injection of cancer cells results in higher rates of brain metastases. However, it is technically demanding and has high post-operative morbidity and mortality, limiting its use in large preclinical studies [27,28].

In this study, we developed a reproducible surgical method for establishing orthotopic BCBM through intracarotid injection of cancer cells. This method led to low morbidity and mortality rates similar to the stereotactic model. We compared the new intracarotid model with the stereotactic model regarding tumour establishment and growth. Finally, BBB permeability was evaluated in vivo using gadolinium (Gd)-contrasted magnetic resonance imaging (MRI) and ex vivo using fluorescent immunohistochemistry and light sheet microscopy.

## 2. Materials and Methods

### 2.1. Animal Models

BT474 cells (ATCC, #HTB-20, Manassas, VA, USA) were cultured in DMEM: RPMI 1640 (1:1) supplemented with 10% fetal bovine serum (FBS) and 1% penicillin–streptomycin (P/S). MDA-MB-231.Luc2 (Caliper Life Science, Hopkinton, MA, USA) were cultured in Leibovitz’s L-15 medium supplemented with 15% FBS, 1% P/S and 2 mM glutamine. The cells were harvested and resuspended in growth medium without P/S to 4 × 10^6^ cells/mL for intracarotid inoculation and 20 × 10^6^ cells/mL for stereotactic implantation.

All surgeries were performed in female NMRI nude mice at the age of 7 weeks (Janvier, Le Genest-Saint-Isle, France). The number of animals used in each model was determined based on prior in-house data on survival and tumour take rates. Animals were provided with analgesia during surgery and three days after by subcutaneous administration of 20 mg/kg Metacam (Pharmo, #386860, Morristown, NJ, USA). Animals bearing BCBM established by BT474 cells were given estrogen-supplemented water (12.5 µg/mL) to promote tumour growth.

Stereotactic implantation was performed by placing animals in a stereotactic frame. A cut was made on the scalp and the periosteum was removed. A hole was drilled 1.5;1 mm (x;y) respective to the bregma before a 22G Hamilton syringe was inserted 2 mm into the brain. Cell suspensions (10 µL, 0.2 × 10^6^ cells) were injected over five minutes using an infusion pump. Local analgesic (lidocain–bupivacain (1:0.8)) was administrated to the scalp before the incision was closed by suture.

Intracarotid inoculation was performed by placing animals in a supine position and securing the forelimbs to a sterile barrier using tape. A cut was made from the top of the sternum towards the jaw. The common carotid artery (CCA) was trimmed for connective tissue and membranes and ligated at the most caudal position possible. A small cut was made in the CCA to allow the insertion of a catheter containing cell suspension. Cell suspensions (50 µL, 0.2 × 10^6^ cells) were injected as a bolus, while the external carotid artery was momentarily closed using forceps. After injection, the CCA was ligated cranially before the catheter was removed, and the incision was closed by sutures.

Animal welfare was monitored daily for up to seven days after surgery according to Supplementary Table S1.

### 2.2. Magnetic Resonance Imaging

T2-weighted MRI was performed to monitor and characterize intracranial BCBM. MRI was performed weekly starting one week after implantation. Animals were anaesthetized before being placed in a prone position in a heated MRI bed. A surface coil (30 mm i.d) was placed over the head. Images were acquired on a 7.0 T Bruker Pharmascan (Bruker, Karlsruhe, Germany). The following imaging parameters were used:

T2_TurboRARE (repetition time = 2500 ms; echo time = 37.47 ms; echo spacing = 9.369 ms; rare factor = 8; 3 averages; fov: 20 × 20 cm^2^; slice thickness = 0.5 mm; slide gap = 0.00 mm; matrix = 256 × 256; coronal slices n = 15; axial slices n = 27).

Gd-contrasted T1-weighted MRI was performed at study end to evaluate the BBB permeability in vivo. A catheter containing heparinized saline (10 U/mL) was inserted into the lateral tail vein of anaesthetized animals before placing the animal in the preclinical MRI scanner. A baseline T1-weighted MRI scan was performed prior to the injection of 100 µL Gd-contrast (Apotek, Copenhagen, Denmark, #51 38 95) (20 µmol/mL). The following imaging parameters were used:

T1_TurboRARE (repetition time = 500 ms; echo time = 12.29 ms; echo spacing = 6.143 ms; rare factor = 8; 2 averages; fov: 20 × 20 cm^2^; slice thickness = 0.5 mm; slide gap = 0.1 mm; matrix = 256 × 256; coronal slices n = 8; axial slices n = 8).

MRI scans were analysed using Horos Project v.3.3.6. Tumour volumes were extrapolated by drawing regions of interest (ROIs) on coronal and axial slices of individual metastases. Accumulated tumour volumes were calculated by adding individual metastases volumes. Signal intensities were evaluated post Gd-contrast by drawing ROIs on T1-weighted MRI scans. The BBB permeability was evaluated by extrapolating integrated values for metastases and healthy brain tissue and normalizing these to the area.

### 2.3. Bioluminescence Imaging

BLI was performed weekly in animals bearing BCBM established by MDA-MB-231.Luc2 cells with the first imaging session performed four days after surgery.

Intraperitoneal injection of 150 mg/kg D-Luciferin (SynChem, #BC219, Elk Grove Village, IL, USA) was administrated to awaken animals 12 min prior to imaging. Images were acquired on the AMT HT system (Spectral Instruments Imaging, Tucson, AZ, USA). The following imaging parameters were used:

luminescence imaging (exposure time = 1 s; binning = low (2); fstop = 1.2; emission: open; fov = 25; object height = 1.5 cm).

BLI was analysed using Aura64 software.(v.5.0.1) BLS was calculated for individual animals by drawing ROIs on the scans. A background ROI was placed on the lower back of the individual animals, and the BLS was calculated as follows:

BLS = ROI (brain) – ROI (background);

The BLS is presented as radiance (photons per second).

### 2.4. Fluorescent Immunohistochemistry

Metastatic counts and their proliferative potential were investigated ex vivo by fluorescent immunohistochemistry staining for Ku80 (0.03 µg/mL, Cell Signaling Technologies, #2180S, Beverly, MA, USA) and Ki67 (1.5 µg/mL Abcam, #AB15580, Cambridge, UK), respectively, using Cy-3 conjugated Goat anti-rabbit as the secondary antibody (750 µg/mL, Jackson, #111-165-144, West Grove, PA, USA) and the free-floating method. Counter-staining was performed using DAPI (0.05 µg/mL, Thermo Scientific, #815-968-0747, Waltham, MA, USA). BBB permeability was evaluated by injecting 100 µL fluorescein sodium (Sigma-Aldrich, #1.03887, St. Louis, MI, USA) one hour prior to euthanasia.

Animals were perfused with heparinized PBS (10 U/mL) before the brain was isolated and fixed in 4% formaldehyde (48 h at room temperature (RT)), cryoprotected in 30% sucrose solution (4 °C), and stored at −80 °C until sectioning. The brains were serially sectioned on a cryostat at 30 µm thickness before staining.

Antigen retrieval was performed in a citrate buffer (pH = 8.5) for 30 min at 80 °C. Sections were blocked for unspecific binding using a 2% non-fat dry milk solution containing 2% goat serum. Primary antibodies staining was performed at 4 °C overnight, while secondary staining (Jackson, #111-165-144) and DAPI was performed at RT for 1 h. Tissue sections were mounted on TOMO glass slides before being covered by cover glass using HIGHDEF^®^ IHC fluoromounting (AH Diagnostics, #ADI-950-260-0025, Tilst, Denmark). Mounted sections were images using VS200 slide scanner (Evident, Tokyo, Japan), and images were analysed using OlyVIA (v.4.1).

### 2.5. Light Sheet Microscopy

A subset of animals was included for LSM visualizing brain metastases, BBB permeability, and the blood vessels within the brain.

One hour prior to euthanasia, animals were injected with 100 µL fluorescein sodium, as described above, as well as 100 µL (0.1 mg/mL) wheat Germ Agglutinin fluorescently labelled with AF-647 (Thermo Fischer Scientific, #W32466, Waltham, MA, USA).

Animals were euthanized by perfusion with heparinized PBS followed by perfusion with 4% formaldehyde using an infusion pump. Brains were carefully isolated, fixated in 4% formaldehyde at 4 °C overnight, and stored in PBS at 4 °C until further processed. Clearing of fixated brains were performed following the PEGASOS protocol [29]. In short, brains were decolorized using 25% Quadrol, stained with Sytox orange (Thermo Fisher Scientific, #S11368, Mumbai, India), delipidated in a series of increasing tert-butanol concentration series, dehydrated using tB-PEG, and finally cleared using Eci. The entire clearing and staining process took 10 days. Images were obtained using Zeiss LS7 (Carl Zeiss Microscopy GmbH, Jena, Germany) with data analysis performed using Huygen professional (v.24.10) and Imaris (v.10.2.0).

### 2.6. Statistical Analysis

All data are presented as mean ± SEM unless otherwise stated. Animals that did not develop a tumour after inoculation were excluded from any further analysis regarding tumour number, growth and BBB permeability. Statistical analyses, comparing body weight loss and BBB leakiness, were performed using Student’s *t*-test with *p*-values ≤ 0.05 reported as statistically significant. Survival is presented in Kaplan–Meier curve with each datapoint representing animals being euthanized based on humane endpoints. Data is processed and presented in GraphPad Prism (v.10.1.1, San Diego, CA, USA).

## 3. Results

### 3.1. Establishing BCBM by Stereotactic and Intracarotid Inoculation

**Figure 1:**
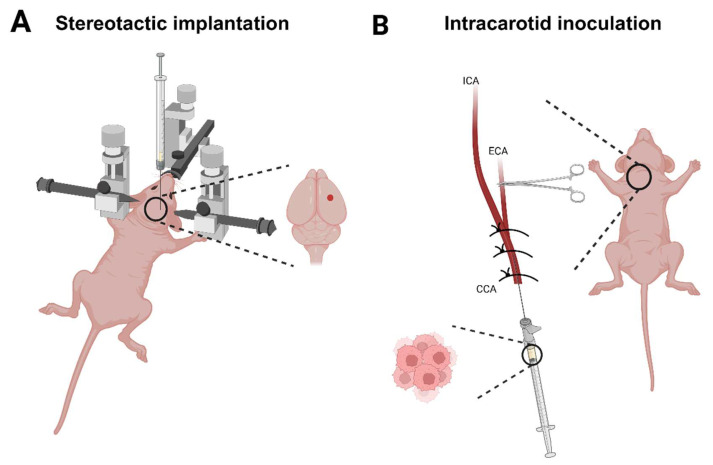
Methods for establishing BCBM in NMRI nude mice. (**A**) Stereotactic implantation was performed in anaesthetised animals placed in a stereotactic frame. A small hole was drilled 1.5 mm right and 1 mm below bregma, and cells were slowly injected 2 mm into the brain using a syringe pump. (**B**) Intracarotid inoculation was performed in anaesthetised animals placed in a supine position. The CCA was isolated and dissected before cells were injected using a catheter. During cell injection, the ECA was closed using a pean forceps. The figure was created in https://BioRender.com. BCBM: breast cancer brain metastases, CCA: common carotid artery, ECA: external carotid artery, ICA: internal carotid artery.

To compare the two models of BCBM in terms of tumour establishment and animal welfare, BCBMs were established in NMRI nude female mice using human breast ductal carcinoma (BT474 (HER2 3+)) and adenocarcinoma cell lines (MDA-MB-231.Luc2 (TNBC)) by stereotactic implantation (Figure 1A) and intracarotid inoculation (Figure 1B). Stereotactic implantation was performed within 10 min per animal, while intracarotid inoculation was performed within 20 min per animal.

To support the use of an intracarotid model for BCBM in larger preclinical studies, the model must demonstrate similar success rates as the stereotactic model, which is generally reported to have high tumour take rates and post-surgery survival. Several optimisations of the surgical procedure for intracarotid inoculation were conducted before a low animal mortality and high tumour take rate were achieved.

The first optimization of the intracarotid method evaluated the success rate of post-surgery survival and tumour establishment in animals inoculated using an insulin syringe (31 G) and a mouse carotid catheter.

Intracarotid inoculation using an insulin syringe led to surgical trauma (tearing of the carotid artery) and backflow of cell suspension into the neck cavity, resulting in tumour formation at the inoculation site. Using a mouse carotid catheter, limited surgical trauma was inflicted, with no incidence of backflow observed. Based on these observations, the following optimisations were performed using a mouse carotid catheter.

The carotid artery can be divided into three main components, including the common carotid artery (CCA), which splits into the external carotid artery (ECA) and the internal carotid artery (ICA). The branching of the CCA into the ECA and ICA is located in close proximity to critical nerve bundles, including the vagus nerve, which is involved in heart rate, respiration, and other fundamental body functions. Thus, inoculation of cells into the CCA, furthest away from the vagus nerve, was performed to decrease the risk of interfering with the vagus nerve. CCA inoculation led to post-surgery survival rates of 100%, but tumour establishment in the nasal and oral cavity were observed with limited BCBM.

As the ECA supplies these regions, we next sought to close it during inoculation. Permanent ligation of the ECA led to severe post-surgery seizures and body weight loss, most likely due to the ligation of nerve endings in close proximity to the ECA. Instead, great efforts were made to trim the ECA by blunt dissection using a forceps, ensuring it was free from nerve bundles before closing it momentarily using pean forceps. Using this procedure, BCBM was established without the formation of extracranial tumours as well as nasal and oral tumours while ensuring fast animal recovery.

### 3.2. High Survival Rates and Animal Welfare in Both Models

A total of 15 animals were included for stereotactic implantation of BT474 cells, while 23 animals were included for intracarotid inoculation of BT474 cells.

**Figure 2:**
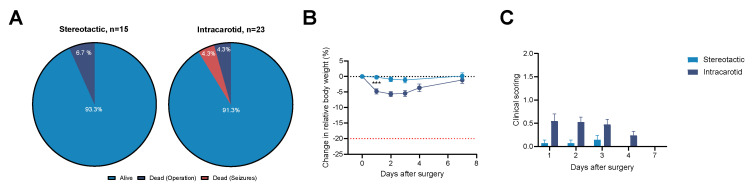
Post-surgery morbidity and mortality in animals following stereotactic and intracarotid inoculation. (**A**) Post-surgery survival rates for animals inoculated by the stereotactic or intracarotid method were reported to be >90%. (**B**) Intracarotid inoculation led to a significant drop in relative body weights on study day one when compared to animals undergoing stereotactic implantation (*p* = 0.003). Seven days after surgery, no significant difference in relative body weights was observed for the two models (*p* = 0.41). (**C**) Initially, intracarotid inoculation gave rise to higher scoring of animals when compared to the stereotactic-implanted animals. The score gradually declined in the days after surgery, and by day seven, a score of 0 was given to all animals, confirming full recovery. Bars represent mean ± SEM (n = 14–21), *** *p* < 0.001. SEM: Standard error of mean.

The post-surgery survival rate was 93.3% and 91.3% for stereotactic implantation and intracarotid inoculation, respectively. Mortality was associated with surgical complications as well as post-operative seizures, as shown in Figure 2A.

Animal welfare and survival were monitored closely for up to one week after surgery with daily body weights and clinical scoring, as shown in Supplementary Table S1. A drop in the relative body weights was observed following both inoculation methods. Stereotactic implantation led to a relative weight loss of −0.2 ± 0.5% on the day after surgery, while intracarotid inoculation led to a significantly larger body weight loss of −4.8 ± 0.8% (Student’s *t*-test: *p* = 0.003). No significant difference in relative body weights was observed one week after surgery between the two models (*p* = 0.41), as shown in Figure 2B.

Clinical scoring of animals showed a small decline in animal welfare in the days after surgery for both models. Animals undergoing stereotactic implantation received a maximum mean score of 0.14 ± 0.1 three days after implantation. A maximum mean score of 0.5 ± 0.2 was given to intracarotid-inoculated animals one day after surgery. Besides one animal in the intracarotid group receiving a score of 3, no animals were given a score above 1 for both models, suggesting minimal animal distress and pain. All animals that were subjected to stereotactic implantation received a score of 0 four days after surgery, while intracarotid-inoculated animals all received a score of 0 seven days after surgery, indicating full recovery from the surgeries, as shown in Figure 2C.

The results showed that the optimised intracarotid inoculation method gave rise to similar high post-surgery survival rates (>90%) and animal welfare as the widely used stereotactic model. This validates the potential use of the intracarotid model in preclinical studies of BCBM.

### 3.3. MRI Revealed Differences in Tumours Established by Stereotactic and Intracarotid Inoculation

Longitudinal imaging was performed to monitor the establishment and growth of intracranial BCBM. BCBM established with BT474 cells was monitored weekly by T2-weighted MRI, starting seven days after surgery. In T2-weighted MRI, hyperintense regions reflect increased water content typically associated with tumour or necrotic tissue, whereas hypointense areas indicate signal loss caused by blood product breakdown often seen in hemorrhage.

**Figure 3:**
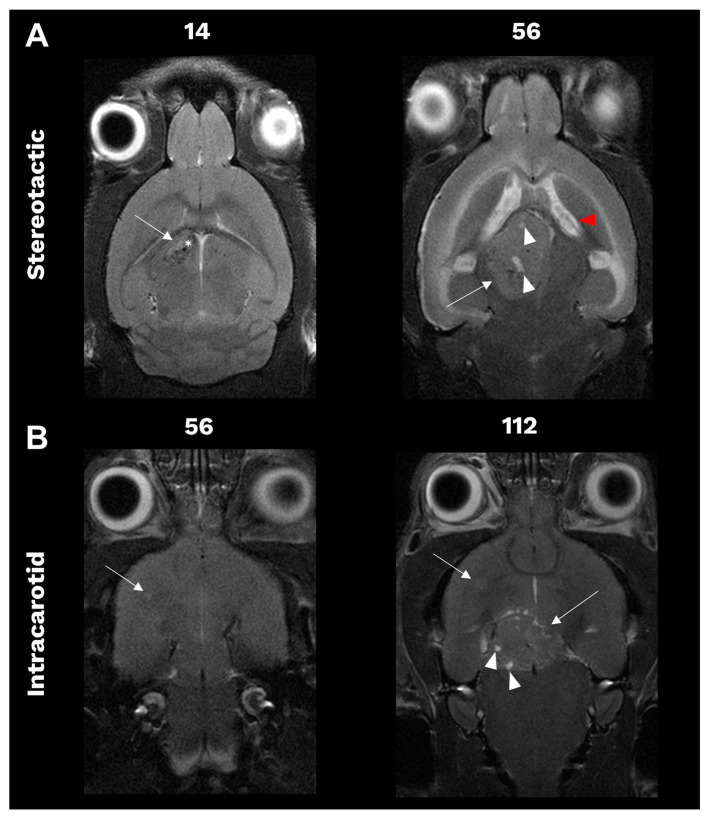
Representative images of animals bearing BCBM (arrows) established by stereotactic implantation (**A**) or intracarotid inoculation (**B**) of BT474 cells at different days after surgery indicated above the images. Intratumoural bleeding (asterisks) was observed in early time points in tumours established by stereotactic implantation. Necrotic areas (white arrowheads) occurred in larger tumours independent of the model, whereas internal hydrocephalus (red arrowhead) only occurred in animals bearing stereotactic-implanted BCBM. BCBM: breast cancer brain metastases, MRI: magnetic resonance imaging.

Stereotactic implantation of BT474 cells led to the formation of a single BCBM at the location corresponding to the implantation site. The tumours were delineated from normal brain tissue as hyperintense areas having clearly defined tumour borders. The first measurable tumours were observed 14 days after implantation with small intratumoural bleedings, appearing as hypointense regions, observed in the early time points. The tumours grew with regular expansile growth with the formation of necrotic areas, presenting as hyperintense areas in larger tumours. Moreover, the increasing tumour mass ultimately led to internal hydrocephalus and oedema, as shown in Figure 3A.

Intracarotid inoculation led to micro-seeding with smaller tumours established throughout the brain. Interestingly, tumours were observed in various anatomic regions such as the cerebral cortex, cerebral nuclei, brain stem and midbrain but confined to the right hemisphere of the brain corresponding to the areas supplied by the right ICA. No tumours were observed in the left hemisphere throughout the study. The tumours established by intracarotid inoculation grew at a slower rate, with the first tumour observed seven weeks after inoculation. The tumours had similar MR signal intensities to BCBM established by stereotactic implantation with clear tumour borders and expansile growth patterns. Larger tumours did present with necrotic areas. However, they did not give rise to internal hydrocephalus and oedema at any time during the study despite reaching similar high tumour volumes as the stereotactic-implanted BCBM, as shown in Figure 3B.

### 3.4. The Number of Individual Tumours and Their Growth Depended on the Establishment Method

**Figure 4:**
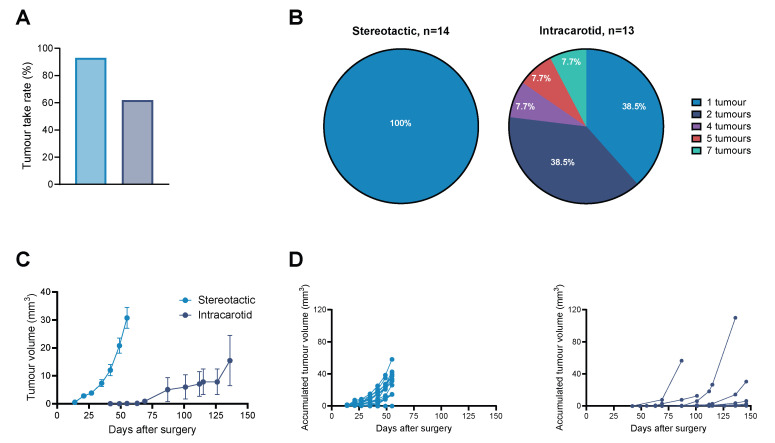
Evaluation of tumour take rate, establishment patterns and growth rate. (**A**) Tumour take rate was found to be 93% for animals bearing stereotactic-implanted BCBM and 62% for intracarotid-inoculated animals. (**B**) Tumour number within each animal was highly conserved for the stereotactic model with the establishment of a single tumour. Intracarotid inoculation led to micro-seeding and establishing one to seven tumours per animal. (**C**) Accumulated tumour growth showed that stereotactic implantation led to faster tumour growth than tumours established by intracarotid inoculation. (**D**) Individual accumulated tumour growth shows homogenous growth in tumours established by stereotactic implantation, whereas intracarotid inoculation led to highly heterogenous tumour growth. Bars represent mean ± SEM (n = 13–14). BCBM: breast cancer brain metastasis, SEM: standard error of mean.

Individual tumour numbers and take rate were highly dependent on the model. Stereotactic implantation led to a take rate of 93%, with only a single tumour established in each animal. Intracarotid inoculation led to a take rate of 62%, with highly diverse metastatic numbers observed between animals, as shown in Figure 4A. The mean metastatic number was 2.4 ± 0.5 tumours per animal, with most animals developing one to two tumours. The maximum number of individual tumours identified by MRI for one animal was seven, as shown in Figure 4B. The tumours were clearly delineated as individual tumours in early establishment. However, some tumours did merge into larger tumours during the study, altering the tumour count and individual volumes.

Individual tumour growth was monitored by drawing regions of interest (ROIs) on T2-weighted MRI images. To compare tumour growth between the two models, accumulated tumour volume was calculated for animals bearing several individual tumours. Mean accumulated tumour growth for the stereotactic and intracarotid model is presented in Figure 4C, with individual accumulated tumour growth presented in Figure 4D. Stereotactic implantation led to fast, homogenous tumour growth with a doubling time of 9.5 ± 0.4 days, while intracarotid inoculation led to slower and more heterogenous tumour growth with a doubling time of 14.4 ± 3.1 days. BCBM established by stereotactic implantation could reach a volume of 58 mm^3^ before causing animal distress, leading to a humane endpoint. The main humane endpoint in animals bearing stereotactic-implanted tumours was body weight loss, often associated with severe hydrocephalus and intracranial pressure.

Intracarotid-inoculated BCBM gave rise to highly diverse tumour growth patterns both between animals and between individual tumours within the same animal. The increase in accumulated tumour volume over time was often driven by one or two tumours experiencing exponential growth, while other tumours in the same animal experienced little or no growth throughout the monitoring period, as shown in Supplementary Figure S1. The tumour location did not seem to be determinant of tumour growth rate. The accumulated tumour volume could reach up to 110 mm^3^ before the animal reached a humane endpoint.

Tumour number and growth are both important parameters to consider in preclinical studies of BCBM. Another crucial factor in tumour growth and treatment response is the BBB. Therefore, we evaluated differences in the BBB leakiness in tumours established by stereotactic implantation and intracarotid inoculation.

### 3.5. Stereotactic Implantation Led to Higher BBB Permeability in Small Tumours

BBB permeability was evaluated in vivo using Gd-contrasted T1-weighted MRI in mice with BT474 tumours.

Stereotactic implantation of cancer cells was performed by inserting a 22 G syringe directly into the brain. To evaluate whether the implantation method itself caused prolonged damage to the BBB, three control animals were submitted to sham stereotactic implantation. Four weeks after surgery, Gd-contrast MRI was performed, with no signs of leaky BBB observed in any of the sham-operated animals, as shown in Supplementary Figure S2.

**Figure 5:**
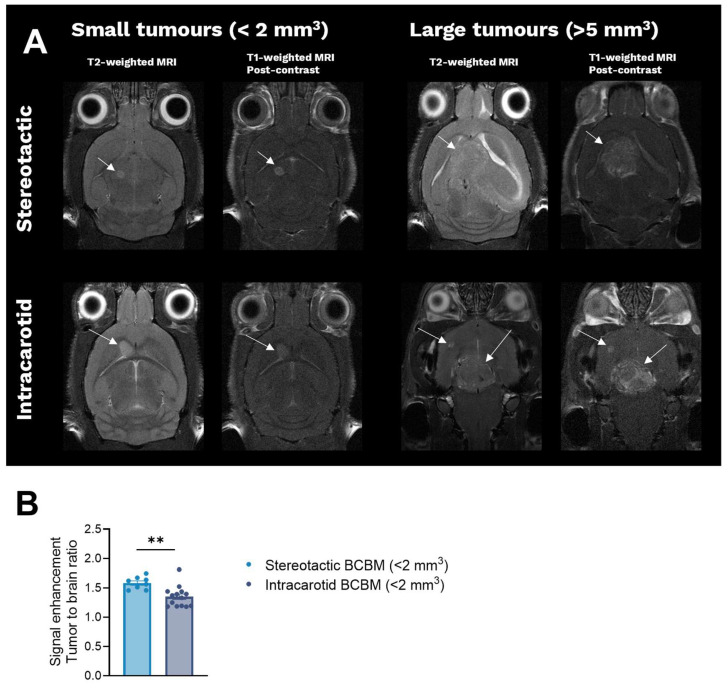
In vivo evaluation of BBB leakiness. (**A**) Representative images of animals bearing BCBM established by stereotactic implantation (top row) and intracarotid inoculation (bottom row) of BT474 cells. T2-weighted MRI was performed to visualise intracranial BCBM (arrows), while Gd-contrasted T1-weighted MRI was performed to evaluate BBB permeability. Hyperintense signal in metastases indicates a leaky BBB. (**B**) Quantitative analysis of BBB leakiness was conducted on small metastases (<2 mm^3^) established by stereotactic implantation and intracarotid inoculation. A significantly higher signal enhancement was observed in smaller metastases established by stereotactic implantation when compared to smaller metastases established by intracarotid inoculation, indicating a leakier BBB in the stereotactic model (Student’s *t*-test, *p* = 0.003). Bars represent mean ± SEM, n = 8–14/group. ** *p* < 0.01. BBB: blood–brain barrier, BCBM: breast cancer brain metastases, MRI: magnetic resonance imaging, SEM: standard error of mean.

BCBM established by both methods gave rise to hyperintense signal directly after Gd-contrast injection, indicating BBB permeability in both models, as shown in Figure 5A. Over time, Gd-contrast accumulated in necrotic areas. Quantitative analysis of signal enhancement in tumours compared to adjacent brain tissue showed that smaller BT474 tumours (<2 mm^3^) established by stereotactic implantation gave rise to significantly higher signal intensities when compared to BT474 tumours established by intracarotid inoculation (Student’s *t*-test, *p* = 0.003), as shown in Figure 5B. These results indicate that BBB permeability was influenced by the method used to establish the BT474 BCBM.

**Figure 6:**
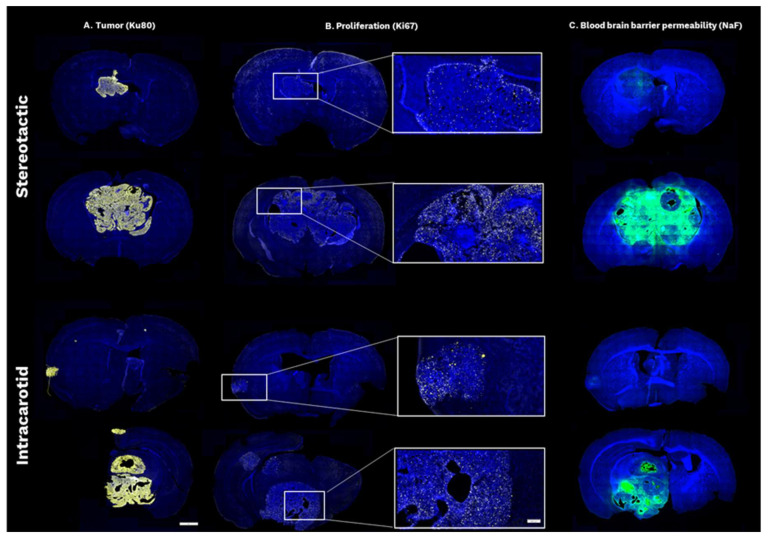
Fluorescent immunohistochemistry of BCBM established by stereotactic (top) and intracarotid (bottom) inoculation of BT474 cells. The top rows represent small tumours, while the bottom rows represent larger tumours. Scale bar is 1 mm and 200 µm on zoomed images. (**A**) The establishment of BCBM was validated ex vivo by fluorescent immunohistochemistry, with human cells visualised by Ku80 staining (yellow). Stereotactic implantation led to the establishment of a single BCBM, whereas intracarotid inoculation led to the formation of several individual tumours, including micro metastases not identified on T2-weighted MRI scans. (**B**) The proliferative index of each tumour was evaluated by Ki67 staining. All tumours had proliferative cells present, with the highest abundance in the tumour border. (**C**) NaF (green) accumulation in tumours was used to evaluate the BBB permeability ex vivo. While smaller tumours had limited accumulation of NaF in both models, the larger tumours established by stereotactic implantation and intracarotid inoculation had a high degree of NaF accumulation, indicating a leaky BBB. BBB: blood–brain barrier, BCBM: breast cancer brain metastases, NaF: sodium fluorescein, MRI: magnetic resonance imaging.

The establishment of BT474 tumours was validated ex vivo by staining sectioned brain tissue for human cells using Ku80 as a marker, as shown in Figure 6A. Image analysis confirmed in vivo findings with the formation of a single tumour in animals bearing BCBM established by stereotactic implantation, while multiple tumours were observed in intracarotid-inoculated animals. Furthermore, micro metastases (as few as five cancer cells) were observed in the intracarotid model, which were not detected on the final T2-weighted MR images. The proliferative status of the individual tumours was evaluated by staining for Ki67 with all tumours containing proliferative cells mainly confined to the tumour border, as shown in Figure 6B. The BBB permeability was furthermore investigated ex vivo using sodium fluorescein (NaF) as a marker for BBB leakiness. NaF was injected in vivo one hour prior to euthanasia, with the accumulation of NaF in tumours confirming in vivo findings of leaky BBB, as shown in Figure 6C.

**Figure 7:**
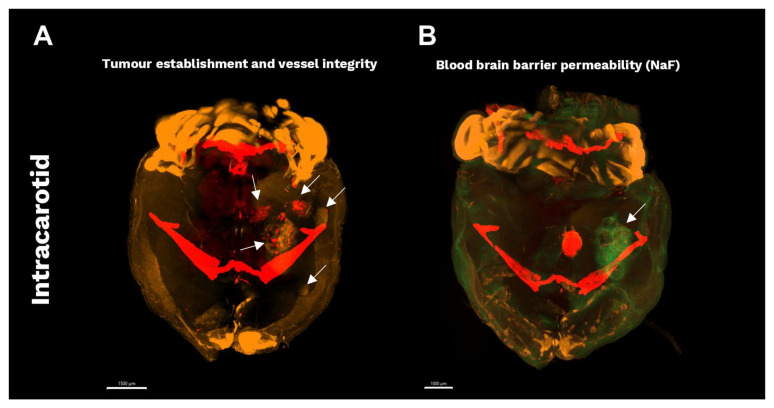
LSM of cleared brains bearing BCBM. (**A**) Intracarotid establishment of BCBM visualized by light sheet microscopy following in vivo staining of blood vessels (red) and ex vivo staining of nuclei (orange). LSM shows multiple metastases (arrows) in the intracarotid model corresponding to tumour number and location observed by MRI. (**B**) BBB permeability was evaluated by in vivo injection of NaF (green), which was found to accumulate in larger tumours, while limited NaF accumulation was observed in smaller tumours. BBB: blood–brain barrier, BCBM: breast cancer brain metastases, LSM: light sheet microscopy, NaF: sodium fluorescein.

The establishment of tumours and the leakiness of the BBB following intracarotid inoculation of BT474 cells were further validated using LSM on cleared brain tissues. Tumours were visualised using Sytox Orange nucleic acid staining, appearing orange. A total of five tumours were detected in the cleared and stained brain, consistent with the number observed by MRI, as shown in Figure 7A. The blood vessels were stained in vivo using AF-647 labelled wheat germ agglutinin, with vessels appearing red. Disintegrated vessels were observed within the tumours, while normal vessel structure was found in the adjacent brain tissue. Finally, BBB leakiness was assessed by in vivo injection of NaF, appearing green, with a leaky BBB observed in the larger tumours, while limited NaF accumulation was found in the smaller tumours, as shown in Figure 7B.

### 3.6. BCBM Was Successfully Established with MDA-MB-231.Luc2 Using Both Methods

To test the reproducibility of the developed intracarotid inoculation method using another tumour model, MDA-MB-231.Luc2 cells (TNBC) were used to establish BCBM as described above.

Stereotactic implantation of MDA-MB-231.Luc2 led to a survival rate of 100% (seven out of seven), while the survival rate was 70% (seven out of ten) for intracarotid inoculation. Intracarotid inoculation led to the euthanasia of animals due to surgical complications and body weight loss. Animals were scored daily to follow surgical recovery, and on study day seven, all animals received a score of 0, indicating full recovery, as shown in Supplementary Figure S3.

While MRI was used to monitor BCBM established with BT474 cells, it was unsuitable for monitoring BCBM established by MDA-MB-231.Luc2 cells as the tumours were diffuse with low tissue contrast, limiting their delineation, as shown in Supplementary Figure S4. Instead, bioluminescence imaging (BLI) was used to monitor tumour establishment and growth allowing monitoring of the entire animal in a few seconds. BLI was performed weekly, starting four days after surgery. Tumour establishment was confirmed when a steady increase in bioluminescence signal (BLS) was observed over three consecutive imaging time points.

**Figure 8:**
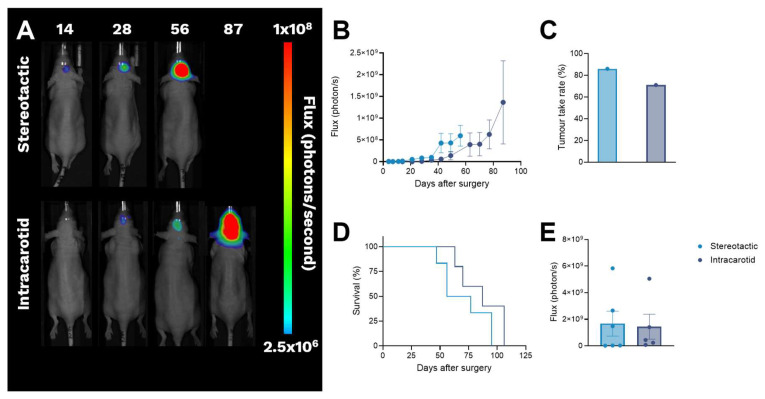
Establishment of BCBM by stereotactic implantation or intracarotid inoculation of MDA-MB-231.Luc2. (**A**) Representative BLI of animals inoculated by the stereotactic (**top**) or intracarotid (**bottom**) method. Stereotactic implantation led to a single BLS focal point corresponding to the implantation site, whereas intracarotid inoculation led to multiple foci in the brain. (**B**) Stereotactic implantation led to faster and more homogeneous tumour growth when compared to the intracarotid-inoculated BCBM. (**C**) Tumour take rates were similar between the stereotactic and intracarotid inoculation, being 86% and 71%, respectively. (**D**) Humane endpoint was reached earlier for animals bearing stereotactic-implanted BCBM (study day 42–96) when compared to animals bearing intracarotid-inoculated BCBM (study day 63–106). (**E**) BLS at euthanasia in animals bearing stereotactic and intracarotid inoculated BCBCM. Bars represent mean ± SEM, n = 5–6. BCBM: breast cancer brain metastases, BLI: bioluminescence imaging, BLS: bioluminescence signal, Flux: photons per second, SEM: standard error of mean.

Stereotactic implantation led to tumour establishment seven days after surgery with a mean BLS of 3.6 × 10^6^ ± 1.7 × 10^6^ photons per second (flux) observed at this time point. Intracarotid inoculation led to tumour establishment 28 days after surgery with tumours presenting with a mean BSL of 1.3 × 10^7^ ± 5.2 × 10^6^ flux. Stereotactic implantation resulted in a single BLS focal point corresponding to the implantation site, whereas intracarotid inoculation led to multiple foci scattered throughout the head, as shown in Figure 8A. A steady increase in BLS over time indicated successful establishment and continuous growth of orthotopic BCBM in both models, as shown in Figure 8B.

A tumour take rate of 86% (six out of seven) was achieved by stereotactic implantation, while the tumour take rate for animals inoculated by the intracarotid method reached 71% (five out of seven), as shown in Figure 8C.

BCBM implanted by the stereotactic method led to humane endpoint between study day 42 and 95, with the mean BLS at the day of euthanasia being 1.7 × 10^9^ ± 9.4 × 10^8^ flux. Intracarotid inoculation led to slower and more heterogeneous tumour growth, with animals reaching a humane endpoint 63–106 days after inoculation, as shown in Figure 8D. The mean BLS at euthanasia in the intracarotid group was 1.4 × 10^9^ ± 8.5 × 10^8^ photons pr second, as shown in Figure 8E. No BLS was observed in any other organ throughout the study for animals inoculated with either of the methods.

## 4. Discussion

Stereotactic implantation of cancer cells is a well-established model for studying BCBM, with high survival and take rates [30]. However, it lacks pathophysiological resemblance to BCBM. In contrast, intracarotid inoculation of cancer cells has been shown to induce metastatic disease resembling BCBM to a greater extent than the stereotactic model but often results in lower post-surgery survival and animal welfare concerns.

In this study, we optimised the intracarotid inoculation method, achieving reduced post-surgery mortality alongside a high incidence of BCBM. Previous work reported that inoculating cancer cells into the CCA led to BCBM with limited animal distress; however, observations of unwanted lung tumours were further reported in this model [31,32]. Our findings diverged from these observations, with tumour development in the nasal and oral cavities and limited establishment of BCBM. To avoid this and to boost BCBM formation, the ECA was closed, directing the cells to the ICA. Permanent ligation of the ECA, as also described by others ([27]), led to significant body weight loss and post-surgery seizures, indicating trauma to the nerve endings located close to the ECA. Instead, temporarily closing the ECA using forceps enabled efficient BCBM establishment while ensuring fast animal recovery.

We evaluated the translational value and applicability of our refined intracarotid inoculation model against the stereotactic model concerning post-surgery survival, tumour growth, and BBB permeability.

Despite optimisation, stereotactic implantation consistently resulted in less animal distress and higher survival rates for both BT474 and MDA-MB-231.Luc2 cell lines, with over 90% survival rates compared to variable outcomes from intracarotid inoculation. This variation underscores the technical skills required for effective inoculation. This observation is true for both inoculation methods, as others have reported survival rates below 50% following stereotactic implantation [23]. The impact of technical advancement on survival rate was further supported by the higher survival rate observed in intracarotid-inoculated BT474 cells (91.3%), which were inoculated later than MDA-MB-321.Luc2 cells (70.0%). Besides enhanced technical skills, the variation in survival rates following intracarotid inoculation could also be cell-line driven, as has been observed by others [27].

Tumour establishment and growth were influenced by the inoculation method. Stereotactic implantation resulted in high tumour take rate (>80%) with the formation of a single tumour, while intracarotid inoculation led to lower tumour take rate (62–71%) with micro-seeding and multiple lesions within a single brain. These observations align with the existing literature on the two models [33,34,35,36]. However, while previous reports indicated high rates of extracranial tumours following intracarotid inoculation, no extracranial tumours were observed following our inoculation method.

The inoculation method further impacted tumour growth, which was particularly pronounced in the BT474 model. Stereotactic implantation led to rapid and homogenous tumour growth, while intracarotid inoculation resulted in slower and more heterogenous tumour growth. Additionally, BCBM established by intracarotid inoculation could reach larger accumulated tumour volumes than stereotactic-implanted BCBM without causing the animal distress usually observed in intracranial tumour-bearing animals at later time points. Distress often correlates with internal hydrocephalus [37], which was not observed in intracarotid-inoculated animals. These findings provide an extended treatment window for preclinical efficacy studies using intracarotid-inoculated BT474 BCBM, enabling the evaluation of compounds with limited risk of tumour escape. Moreover, it allows a longitudinal evaluation of a compound’s ability to induce and maintain a treatment response over a longer duration than is possible with the stereotactic model. Lastly, the slower tumour growth resembles the development of metastatic disease in patients, where tumours develop over a long period of time.

The same difference in tumour growth rate between intracarotid- and stereotactic-inoculated BCBM was not observed for MDA-MB-231.Luc2 tumours. This discrepancy could be cell-line-specific or due to the different monitoring methodologies employed, as MDA-MB-231.Luc2 tumours were monitored using BLI due to MRI being unsuitable for detecting these diffuse tumours. While MRI allows for monitoring of individual tumours and offers high spatial resolution, BLI allows fast and cost-efficient tumour monitoring on a whole-body level. However, several factors impact BLS, including tumour size, tumour location, and substrate delivery, making it less sensitive for accurately monitoring tumour volumes alone [38].

Tumour take rate, number, and growth are important factors to consider when deciding on a model for preclinical evaluation of therapies targeting BCBM. The lower take rate observed in the intracarotid model would necessitate an increased number of animals undergoing intracarotid inoculation to reach a certain population size needed in preclinical studies. The BBB is another essential factor impacting drug delivery and, thus, treatment response.

Different inoculation methods caused varying tumour signal enhancement after Gd-contrast injection, indicating differences in BBB leakiness for BT474 tumours. Stereotactic-implanted BT474 BCBM showed significantly greater contrast enhancement than intracarotid-inoculated BCBM, indicating higher BBB leakiness in the stereotactic model (*p* = 0.003). This increased leakiness could lead to enhanced compound delivery to intracranial lesions, leading to an overestimation of compound efficacy in preclinical studies, with up to 30% overestimation reported, thereby contributing to failures in replicating anti-tumour efficacy in concurrent clinical trials [9]. To evaluate whether signal enhancement was due to surgical intervention, we assessed BBB permeability in sham stereotactic operated animals and found no indications on leaky BBB in these animals, consistent with other studies [24]. These observations indicate that the increased leakiness of the BBB found in stereotactic-implanted BT474 BCBM could be caused by the fast and aggressive tumour growth observed in this model when compared to the slower-growing BT474 BCBM established by intracarotid inoculation. Fast-growing tumours rely on angiogenesis and the formation of new vessels to sustain the increasing demand for oxygen and nutrients for continuous tumour growth and expansion, while dormant and slow-growing tumours do not [39,40]. Newly formed vessels are usually disintegrated and leaky, which could explain the increased BBB leakiness observed in this model. This hypothesis is further supported by LSM observations of abnormal and disintegrated vessels in larger tumours compared to smaller ones.

## 5. Conclusions

While stereotactic implantation of tumours is technically easier with favourable growth rates, our findings indicate that the intracarotid model holds significant translational potential as it presents key pathophysiological parameters for BCBM observed in patients, such as extravasation, micro-seeding, and extended therapeutic window, allowing for proper investigation of novel therapeutic drugs for BCBM. Furthermore, this approach is adaptable for investigating various brain metastases, including those as the deadly and highly prevalent as lung cancer brain metastases. Evaluating changes and interplay between tumour establishment and therapeutic response within the tumour microenvironment would further increase the translational value of the BCBM models described in this paper.

## Data Availability

All raw data are available from the corresponding author upon reasonable request.

## Supplementary Materials

The following supporting information can be downloaded at: https://www.mdpi.com/article/10.3390/cancers18071095/s1, Figure S1: Individual growth of metastasis established by intracarotid inoculation of BT474 cells from the day of surgery (day 0) until study end (day 146). BCBM: breast cancer brain metastases; Figure S2: Representative MR images of animals stereotactically implanted with saline. T2-weighted and Gd-contrasted T1-weighted MRI was used to evaluate permanent BBB damage following stereotactic implantation of saline. The red arrow indicates the site of implantation with no signs of leaky BBB observed four weeks after implantation. BBB: blood–brain barrier, Gd: gadolinium, MRI: magnetic resonance imaging; Figure S3: Post-surgery survival and animal welfare of mice bearing intracranial BCBM established with MDA-MB-231.Luc2 cells; Figure S4: Longitudinal T2-weighted MRI was used to monitor intracranial BCBM established with MDA-MB-231.Luc2 cells, with the day of the scan written above each image. Stereotactic implantation and intracarotid inoculation led to the establishment of diffuse BCBM with little tissue contrast. Metastasis delineation was not possible to perform. BCBM: breast cancer brain metastases, MRI: magnetic resonance imaging; Table S1: Clinical scoring of animals with BCBM. Animals were submitted to daily clinical scoring for up to seven days after surgery. Clinical scoring was used to monitor and ensure animal welfare and was performed by trained animal technicians.

## Abbreviations

The following abbreviations are used in this manuscript:

BBB	Blood–brain barrier
BC	Breast cancer
BCBM	Breast cancer brain metastases
BLI	Bioluminescence imaging
BLS	Bioluminescence signal
CCA	Common carotid artery
ECA	External carotid artery
FBS	Fetal bovine serum
Gd	Gadolinium
HER2	Human epidermal growth factor receptor 2
ICA	Internal carotid artery
LSM	Light sheet microscopy
MRI	Magnetic resonance imaging
NaF	Sodium fluorescein
P/S	Penicillin–streptomycin
ROI	Region of interest
RT	Room temperature
SEM	Standard error of mean
TNBC	Triple-negative breast cancer
