# Supplementary material for: Evaluating the Translation Value of Two In Vivo Models for Breast Cancer Brain Metastases
Source: Cancers (Basel). 2026 Mar 27;18(7):1095. doi: 10.3390/cancers18071095 (PMC13072151; doi:10.3390/cancers18071095)
Supplement: Supplementary file 1 [file cancers-18-01095-s001.zip › cancers-4195180-supplementary.pdf]

cancers MDPI

Supplementary materials

Table S1. Clinical scoring of animals with BCBM. Animals were submitted to daily clinical scoring for up to seven days after surgery. Clinical scoring was used to monitor and ensure animal welfare and was performed by trained animal technicians.

Score	Variable	Stress score	Response
0	No symptoms	None	None
1	Reduced spontaneous activity	Light	Increased observation from once daily to twice daily.
	Lack of grooming		
	Weight loss ≤ 10 %		
	Ocular discharge		
2	Symptoms from score 1 and/or	Moderate	Intervention. Can be pain treatment, fluid therapy, softened food, or other relevant treatment. Observation as a minimum twice daily. This can be increased as required to ensure that score 3 does not occur.
	Reduced unprovoked activity		
	Reduced provoked activity		
	Slightly bristled fur and/or whiskers		
	10 % < Weight loss < 20 %		
	Nasal discharge		
3	Half-closed eyes	Significant	The animal is euthanized
	Weight loss 20 %		
	Reduced water and food intake		
	Bristled fur and/or whiskers		

 https://doi.org/10.3390/cancers18071095

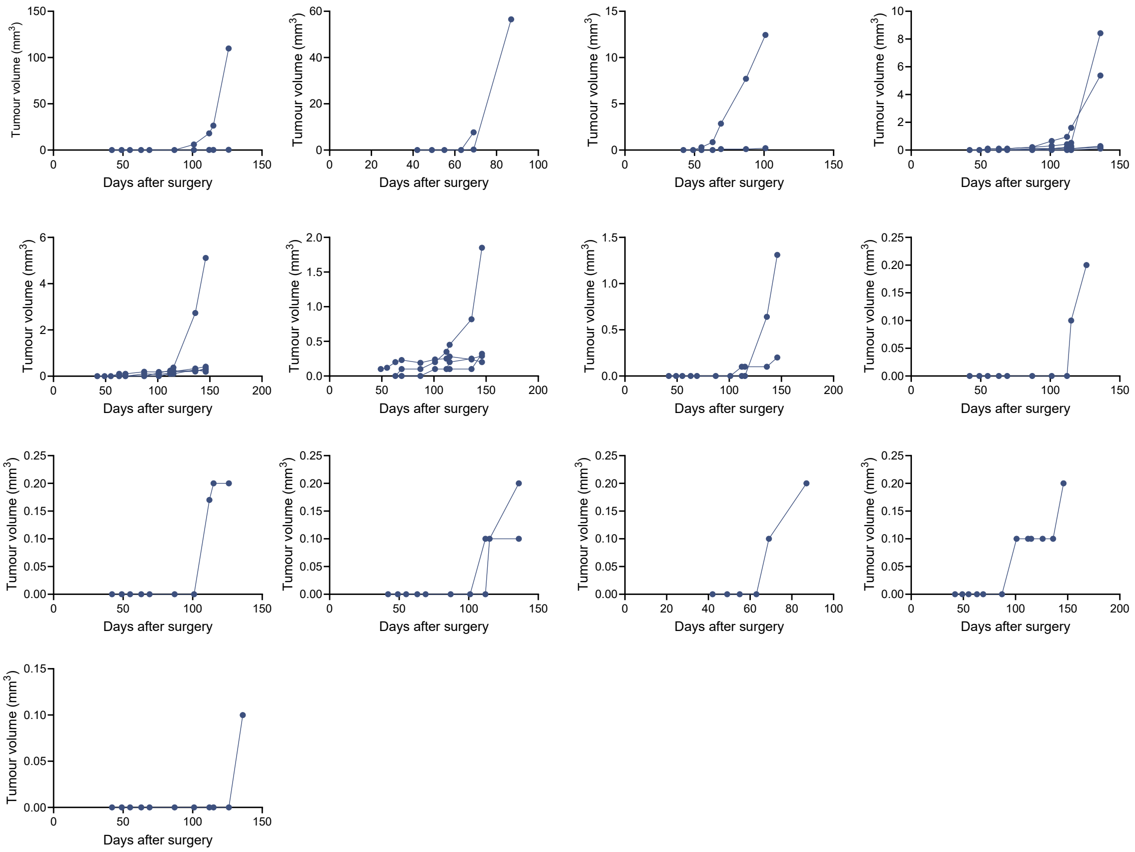

Figure S1. Individual growth of metastasis established by intracarotid inoculation of BT474 cells from the day of surgery (day 0) until study end (day 146). BCBM: Breast cancer brain metastases.

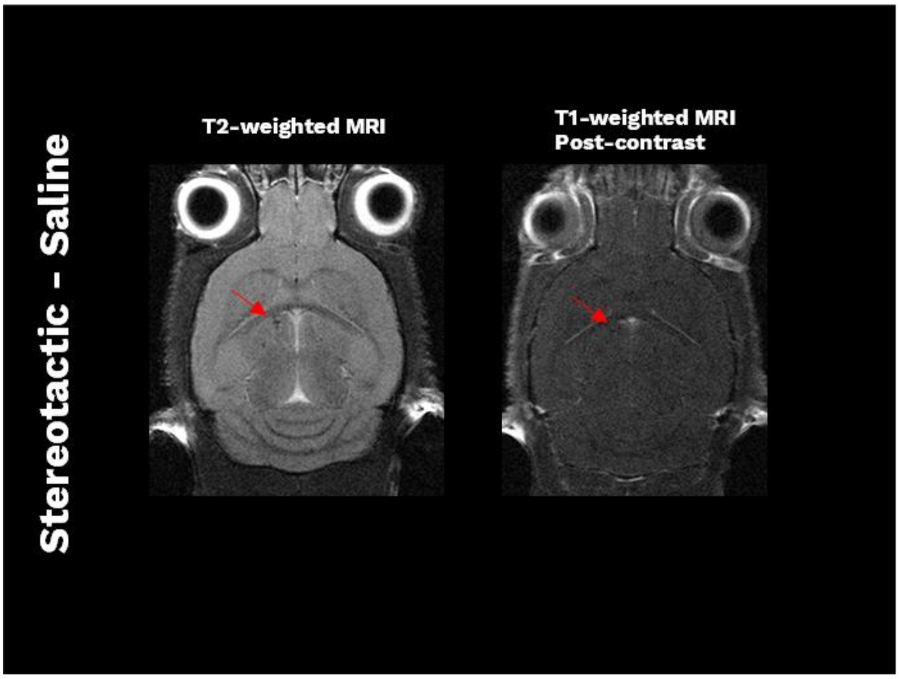

Figure S2. Representative MR images of animals stereotactically implanted with saline. T2-weighted and Gd-contrasted T1-weighted MRI was used to evaluate permenant BBB damage following stereotactic implantation of saline. The red arrow indicates the site of implantation with no signs of leaky BBB observed four weeks after implantation. BBB: Blood brain barrier, Gd: Gadolinium, MRI: Magnetic resonance imaging. .

https://doi.org/10.3390/cancers18071095

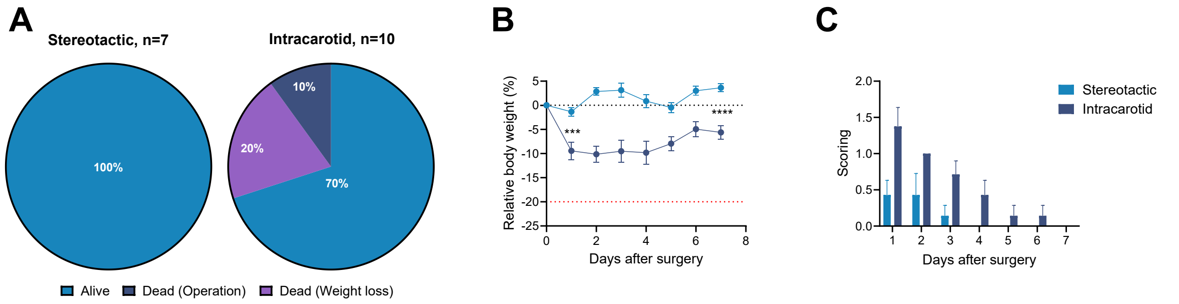

Figure S3. Post-surgery survival and animal welfare of mice bearing intracranial BCBM established with MDA-MB-231.Luc2 cells. (**A**) Survival rate following stereotactic and intracarotid inoculation of MDA-MB-231.Luc2 was 100% and 70%, respectively. (**B**) Animals bearing intracarotid-inoculated BCBM experienced a significant drop in relative weight on the day after surgery when compared to animals bearing stereotactic-implanted BCBM (Student's t-test: p=0.0003). One week after surgery, the intracarotid model maintained to have a significant decreased relative body weight (p<0.0001). (**C**) Animals bearing intracarotid-inoculated BCBM received higher clinical scores throughout the 7-days monitoring period. One week after surgery, all animals were given a score of 0, indicating full recovery from surgery. Bars represent mean ± SEM (n=7-10). ***p<0.001 and ****p<0.0001. BCBM: Breast cancer brain metastases.

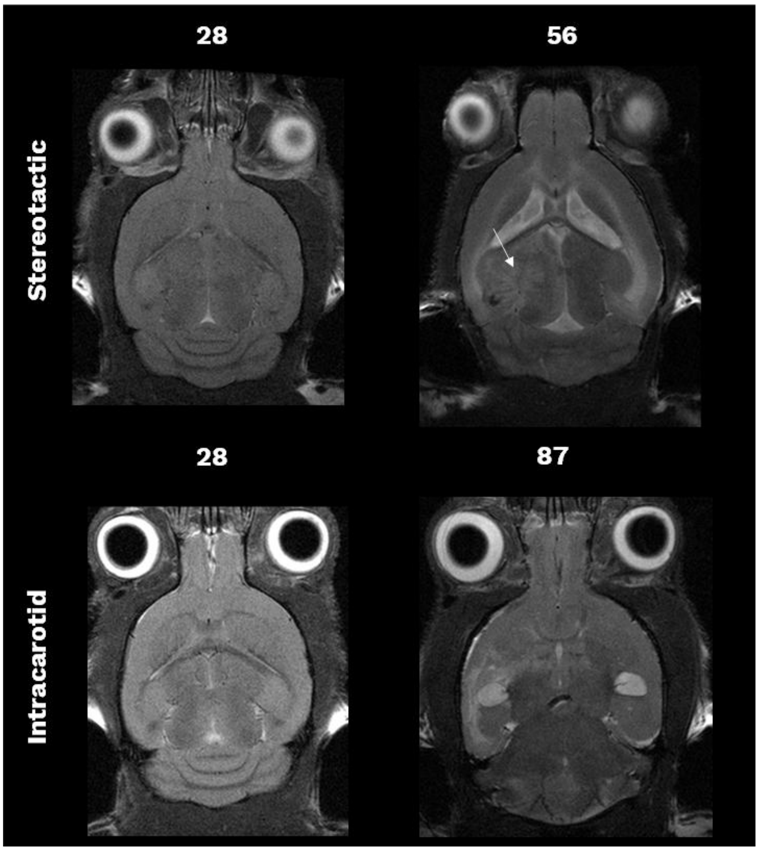

Figure S4. Longitudinal T2-weighted MRI was used to monitor intracranial BCBM established with MDA-MB-231.Luc2 cells with the day of the scan written above each image. Stereotactic implantation and intracarotid inoculation led to the establishment of diffuse BCBM with little tissue contrast. Metastasis delineation was not possible to perform. BCBM: Breast cancer brain metastases, MRI: Magnetic resonance imaging.

https://doi.org/10.3390/cancers18071095
